# Predicting Invasive Fungal Pathogens Using Invasive Pest Assemblages: Testing Model Predictions in a Virtual World

**DOI:** 10.1371/journal.pone.0025695

**Published:** 2011-10-10

**Authors:** Dean R. Paini, Felix J. J. A. Bianchi, Tobin D. Northfield, Paul J. De Barro

**Affiliations:** 1 Ecosystem Sciences, Commonwealth Scientific and Industrial Research Organisation, Canberra, Australian Capital Territory, Australia; 2 Cooperative Research Centre for National Plant Biosecurity, Canberra, Australian Capital Territory, Australia; 3 Ecosystem Sciences, Commonwealth Scientific and Industrial Research Organisation, Brisbane, Queensland, Australia; 4 Department of Entomology, Washington State University, Pullman, Washington, United States of America; University of Medicine & Dentistry of New Jersey - New Jersey Medical School, United States of America

## Abstract

Predicting future species invasions presents significant challenges to researchers and government agencies. Simply considering the vast number of potential species that could invade an area can be insurmountable. One method, recently suggested, which can analyse large datasets of invasive species simultaneously is that of a self organising map (SOM), a form of artificial neural network which can rank species by establishment likelihood. We used this method to analyse the worldwide distribution of 486 fungal pathogens and then validated the method by creating a virtual world of invasive species in which to test the SOM. This novel validation method allowed us to test SOM's ability to rank those species that can establish above those that can't. Overall, we found the SOM highly effective, having on average, a 96–98% success rate (depending on the virtual world parameters). We also found that regions with fewer species present (i.e. 1–10 species) were more difficult for the SOM to generate an accurately ranked list, with success rates varying from 100% correct down to 0% correct. However, we were able to combine the numbers of species present in a region with clustering patterns in the SOM, to further refine confidence in lists generated from these sparsely populated regions. We then used the results from the virtual world to determine confidences for lists generated from the fungal pathogen dataset. Specifically, for lists generated for Australia and its states and territories, the reliability scores were between 84–98%. We conclude that a SOM analysis is a reliable method for analysing a large dataset of potential invasive species and could be used by biosecurity agencies around the world resulting in a better overall assessment of invasion risk.

## Introduction

While invasive species cause significant environmental and economic damage worldwide [1], [2], [3], predicting which of the hundreds or thousands of potentially invasive species are most likely to invade a region presents a significant challenge. Those tasked with the responsibility of preventing biological invasions are often required to prioritise across often extensive lists of potential invaders as part of resource allocating activities. To facilitate this process, models have been developed and are used, but while some methods have the ability to evaluate the likelihood of invasion or establishment for multiple species simultaneously [4], [5], the vast majority can only assess a single species at a time (e.g. [6], [7], [8], [9], [10]). As a consequence, many biosecurity agencies around the world utilise consultative processes aimed at eliciting expert opinion from researchers, government officers, and industry stakeholders. These experts are often asked to assess and prioritise across a large number of potential invasive species, and while their experience and knowledge may be extensive, it is unlikely to extend to all species under consideration. In addition, this elicitation process can be susceptible to framing, context dependence and motivational bias which can lead to flawed prioritisations, poor decision making and misallocation of usually limited mitigation resources [11].

Recently, a self organising map (SOM) has been used to analyse invasive pest assemblages (IPA) using the presence/absence data of multiple invasive species [12], [13]. A SOM is a type of artificial neural network, which identifies patterns of association amongst invasive species, whereby regions with similar suites of invasive species are clustered and a region-specific likelihood of establishment index (a value between 0 and 1) for each species is generated. The invasive pest assemblage (IPA) present in a region captures a significant proportion of biological, ecological, and abiotic factors that cannot be measured. If two regions have a similar IPA they are likely to have similar characteristics and any species present in one of these regions is likely to be able to establish in the other. The SOM is able to make a similar assessment to the example above except with multiple regions at the world scale. By clustering regions based on assemblages the SOM is able to generate a value (a neuron weight) for each species that indicates the strength of association of that species with a region's species assemblage. This value can then be used as a likelihood of establishment index. It should be made clear that the invasion process involves two steps, arrival and establishment. By analysing species associations, the SOM is only assessing establishment likelihood and is unable to estimate arrival likelihood.

SOM has been used to analyse the worldwide distribution of hundreds of species simultaneously [12] and this approach has been found to be robust to significant errors in the data [14], which are inevitable in such a large dataset. The benefit of a SOM analysis is that it provides a complimentary alternative to the elicitation process and can be used to cross check and challenge expert opinion, which can improve the quality and accountability of the opinions offered [11]. However, thorough model validation is essential in assessing the utility of the SOM approach and the accuracy of the species rankings it generates. For a worldwide data set it may be possible to analyse the historical worldwide species distributions, if they were available, and compare the predicted rankings to the subsequent invasions. However, a mismatch between predictions and invasion would not necessarily indicate a poor model, but simply an inability of the agent to find a suitable pathway that connects the species to the region under consideration.

To address this issue we developed a novel alternative that does not have this disadvantage. We created a “virtual world” filled with invasive species, to simulate the real world. In such a virtual world the regions in which an invasive species is able to establish would be known, and it would then be possible to test the rankings made by a SOM analysis.

There were two main aims of this study. The first was to complete a SOM analysis of the IPA of a worldwide distribution of plant fungal pathogens, thereby generating likelihood lists of establishment for Australia and its states and territories. The second was to validate these predictions using the virtual world approach. We used a dataset of the worldwide distribution of fungal pathogens, and then created a virtual world of the same size (same number of regions and species) within which to validate the predictions and determine the level of confidence for any list generated from the dataset.

## Results

### SOM analysis of fungal pathogen dataset

Establishment likelihood lists of the top 100 absent species were generated for all states and territories of Australia, as well as Australia as a whole (Tables S1, S2, S3, S4, S5, S6, S7, and S8).

### Comparison of virtual world with fungal pathogen dataset

**Figure 1:**
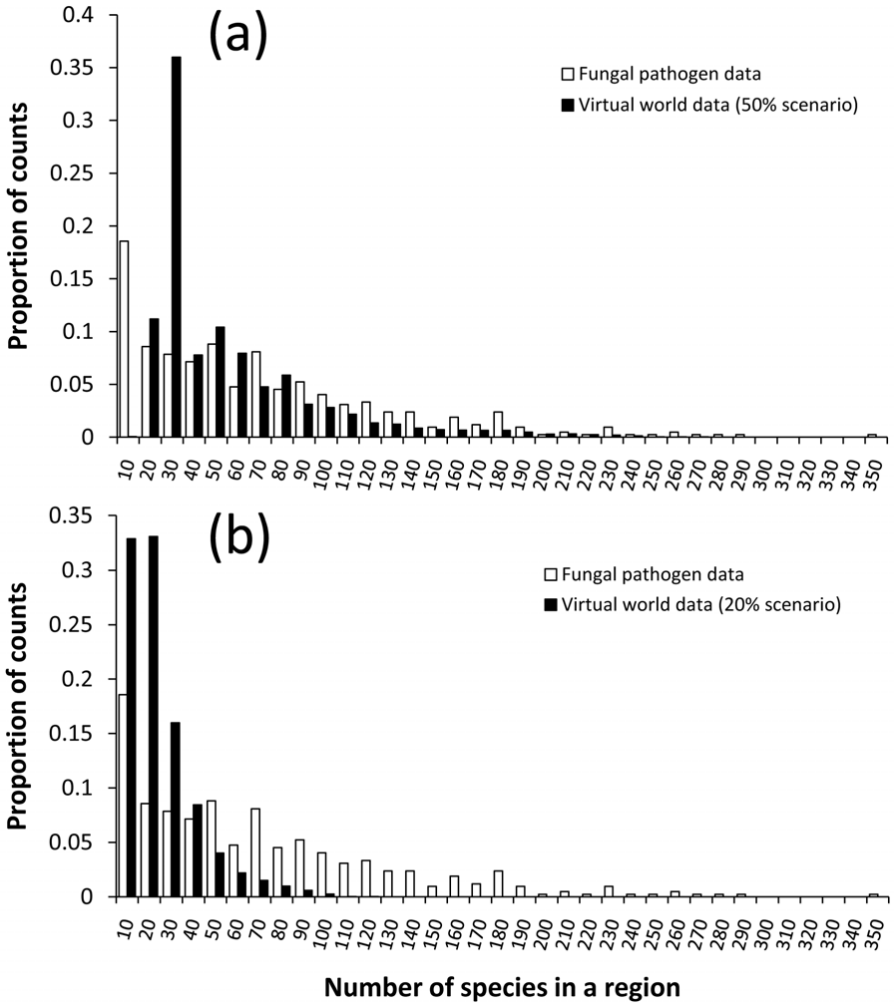
Species Distributions. Comparison of the distribution of species in regions between the original fungal pathogen data set and (a) 100 virtual worlds with 50% distribution, (b) 100 virtual worlds with 20% distribution.

The negative binomial distribution generated from the 20% scenario did not explain the fungal pathogen data set as well as the distribution generated from the 50% scenario (Δ AIC = 1972.51), suggesting that the 50% scenario was a better fit to the fungal pathogen data than the data from the 20% scenario (Figure 1).

### SOM predictive accuracy

**Figure 2:**
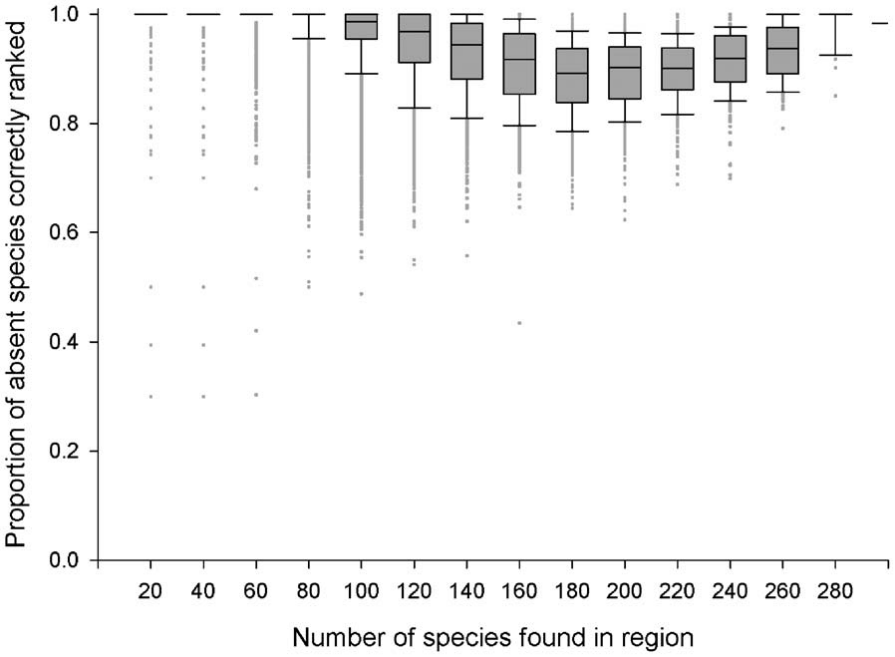
SOM Success Rate. The ability of SOM to successfully rank absent species that can establish in a region above those species that can't establish, as a function of the number of species found in a region. 100 virtual worlds with 50% distribution.

**Figure 3:**
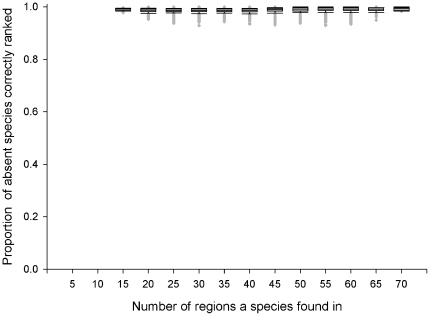
SOM Success Rate. The ability of SOM to successfully rank absent species that can establish in a region above those species that can't establish, as a function of the number of regions a species was found. 100 virtual worlds with 50% distribution.

For 100 virtual worlds, when invasive species were distributed to 50% of their potential distribution (scenario 1), mean success rate was 0.98 (i.e. on average, the SOM correctly ranked 98% of the species that could establish in a region above those that couldn't), though this varied depending on the number of species present (Figure 2). The SOM success rate for species across regions was equally high at 0.99. On average, a species was correctly ranked in the ‘top half’ (if it could establish) or the ‘bottom half’ (if it couldn't establish) of a region's list for 99% of regions, and this varied little with how widely spread the species was (Figure 3).

**Figure 4:**
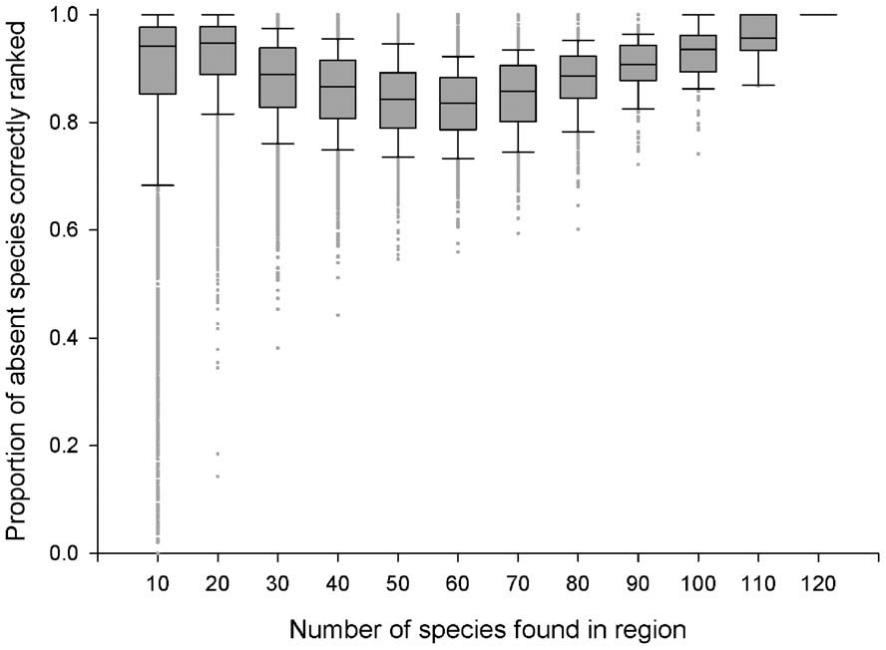
SOM Success Rate. The ability of SOM to successfully rank absent species that can establish in a region above those that can't, as a function of the number of species found in a region. 100 virtual worlds with 20% distribution.

**Figure 5:**
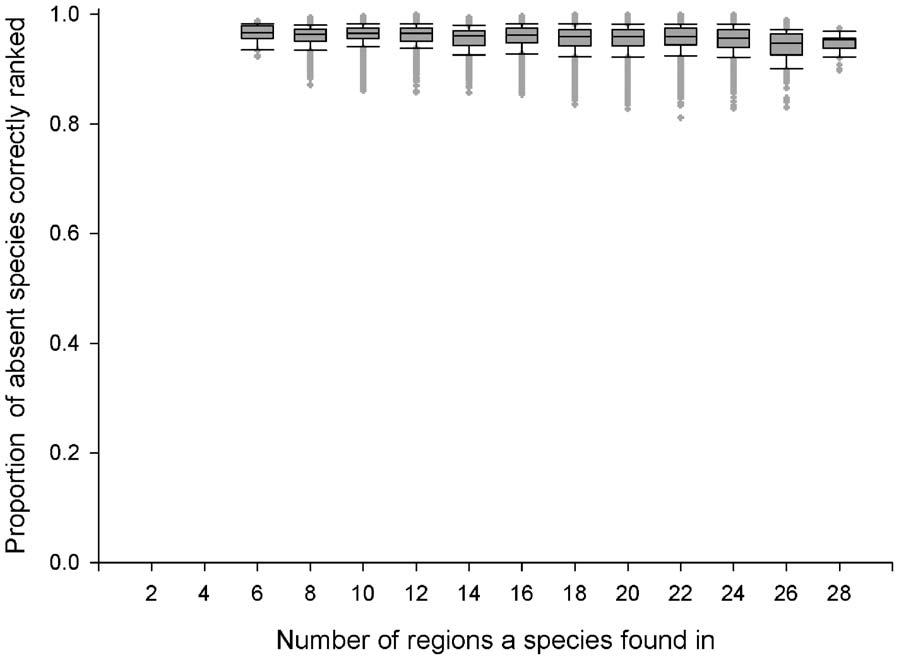
SOM Success Rate. The ability of SOM to successfully rank absent species that can establish in a region above those that can't, as a function of the number of regions a species was found. 100 virtual worlds with 20% distribution.

For another 100 virtual worlds, invasive species were distributed to 20% of their potential distribution. There was greater variation than the 50% scenario (Figure 4), but the overall mean SOM success rate for regions was still high at 0.89. The SOM success rate for species across regions was 0.96 and varied little with how widely spread the species was (Figure 5).

The results presented in Figures 2 and 4 were then used to estimate the confidence in the fungal pathogen lists generated for Australia and its states and territories (Table 1). The lowest SOM success rate (0.84) was for regions with a similar size to Northern Territory (41–50 species present), while the highest SOM success rate (0.98) was for regions with a similar size to Australia (241–250 species present).

**Table 1:** Expected SOM success rates for lists generated for each state and territory of Australia, and Australia as a whole.

Region	No. of species present	VW distribution	SOM success*
Northern Territory	42	20%	0.84
Tasmania	97	20%	0.93
Victoria	111	50%	0.92
Western Australia	112	50%	0.92
South Australia	119	50%	0.92
Queensland	171	50%	0.89
New South Wales	182	50%	0.89
Australia	249	50%	0.98

Success rates drawn from the testing of SOM in 100 virtual worlds with either a 20% or a 50% species distribution. Because a 20% species distribution resulted in regions with less than 110 species, this data could not be used for estimating regions in the fungal pathogen data set containing more species. For these regions, we used the SOM success rates from the 50% species distribution.

*SOM success rates estimated from Figures 2 and 4.

### Regions with 1–10 species

When species were distributed to only 20% of their potential ranges (scenario 2) there was a substantial amount of variation in SOM success rate for regions which only had 1–10 species (Figure 4). We assessed whether the SOM success rate was affected by which neuron in the SOM a region was allocated to (Table S9). While the SOM success rate tended to be reduced for regions that had only a few species, this could be mitigated if that region was found in the same neuron as other regions (Table 2). For example, if a region had only 5 species, then we would have high confidence in a SOM generated risk list only if it was allocated into a neuron with 5 other regions (6 regions in total).

**Table 2:** The interaction between the number of species found in a region and the number of regions allocated to a neuron with regard to SOM's ability to rank species that can establish over those that cannot.

# regions	# of species found in a region									
in a neuron	1	2	3	4	5	6	7	8	9	10
1	0.07	0.34	0.39	0.48	0.55	0.60	0.73	**0.82**	**0.87**	**0.90**
2	0.06	0.42	0.46	0.55	0.64	0.67	0.79	**0.83**	**0.86**	**0.85**
3	0.25	0.50	0.52	0.58	0.63	0.73	0.78	**0.83**	**0.86**	**0.85**
4	0.33	0.33	0.39	0.64	0.77	**0.80**	**0.85**	**0.88**	**0.89**	**0.89**
5		0.71	0.65	0.73	0.78	**0.82**	**0.84**	**0.87**	**0.88**	**0.91**
6			0.50	0.77	**0.85**	**0.84**	**0.86**	**0.88**	**0.90**	**0.90**
7	0.55	**0.84**	0.65	**0.87**	**0.83**	**0.88**	**0.88**	**0.90**	**0.90**	**0.92**
8		0.76	**0.82**	**0.87**	**0.86**	**0.89**	**0.89**	**0.92**	**0.91**	**0.93**
9			**0.97**	**0.94**	**0.94**	**0.93**	**0.94**	**0.93**	**0.93**	**0.92**
10			0.69	**0.90**	**0.88**	**0.93**	**0.91**	**0.93**	**0.92**	**0.94**
11		0.77	**0.86**	**0.89**	**0.94**	**0.92**	**0.95**	**0.95**	**0.96**	**0.94**
12				0.75	**0.89**	**0.92**	**0.93**	**0.94**	**0.94**	**0.93**
13				**0.96**	**0.95**	**0.93**	**0.94**	**0.94**	**0.93**	**0.94**
14				**1.00**	**0.97**	**0.96**	**0.96**	**0.95**	**0.95**	**0.94**

Only those categories in which SOM success rate was above 0.80 (80%) are in bold. It is only these categories in which confidence in the SOM generated likelihood list can be obtained. Data extracted from 100 virtual worlds, with species distributed to 20% of their potential range.

## Discussion

The SOM performed well in the virtual world of invasive species and was able to consistently rank a high percentage of those species that could establish in a region above those that couldn't. Although there could be less confidence in lists generated for those regions with less than ten species present, we can increase this confidence by determining the number of other regions allocated to the same neuron. These results enable significant confidence in any lists generated from a SOM analysis of invasive species and specifically, for the fungal pathogen lists presented here for Australia and its states and territories.

Although the AIC test revealed that the 50% scenario was a closer fit to the fungal pathogen data, the 20% scenario is a more challenging test of the SOM's predictive ability. The 50% distribution has a smaller proportion of regions with 1–10 species, and a larger proportion of regions with 21–30 species, than the fungal pathogen data set (Figure 1a). Regions that hold few species are more challenging for the SOM to correctly distinguish between those species that can establish and those that cannot. Examining the 20% scenario (Figure 1b) reveals a large proportion of regions with only a few species (1–10, or 11–20 species). This would make it more difficult for SOM to predict establishments from this scenario than the fungal pathogen data set. The 20% scenario therefore represents a more conservative test of the SOM predictions and one that we shall consider in more detail.

Generally, we found the ability of the SOM to rank those species that could establish in a region above those that could not, to be very high. On average, 89% of species that could establish in a region were correctly ranked above those that were unable to establish. Only in regions with 1 to 10 species did the SOM analysis have some difficulty, with success rates ranging from 0 to 1. However, within this group, we have been able to identify a characteristic that further refines interpretation of the results (i.e. the number of other regions allocated to the same neuron). The regions allocated to a neuron all have similar pest assemblages and it is this information that is reflected in the likelihoods. If a region has only a small number of species present, the amount of species association information captured appears to be a limiting factor for correct assignment. The presence of additional regions with similar species assemblages provides more associational information and enables more accurate SOM predictions.

We can now use the results of these virtual world tests (specifically, Figures 2 and 4) to estimate our confidence in the lists generated from the SOM analysis of the fungal pathogen dataset, based on the number of species in the region (Table 1). Overall, SOM success rate in the virtual world for regions of similar size to Australia and its states and territories range between 0.84 and 0.98 and provide substantial levels of confidence in the lists generated from the fungal pathogen data set. In addition, we can determine our confidence in any other region's list, including those regions with only 1–10 species (Table S10).

It is interesting to note that across 20% and 50% scenarios the ability of SOM to rank a species was not related to the number of regions a species was found in (Figures 3 and 5), and the SOM success rate was very high (mean: 96–98%). Essentially, the SOM analysis only makes a small number of errors, but these tend to be concentrated in those regions with only 1 to 10 species. The number of regions a species is found in is therefore not related to the SOM predictive power and those species which are poorly distributed are just as well predicted as widespread species.

While the data analysed is that of historical invasions, the opening up of new pathways in the future may lead to new introductions [15], which could alter species associations and SOM species rankings. However, it should be noted that the high success rate of the SOM analysis in its predictive rankings are in the absence of future pathways and subsequent species associations. While these future species associations may provide more information and improve SOM predictive powers, the high predictive power of SOM, even at the conservative 20% scenario, may mean that further information may only slightly improve predictive rankings. Despite this, future work may need to ‘isolate’ a region or regions in the virtual world before, restricting the number of species that can be found there initially. Allowing subsequent invasions into this region would further test SOM predictive powers. However, we would argue that this would be similar to the situation that has arisen in the virtual worlds presented here, whereby some regions only contained a few species. Despite the limited information in these regions, and without allowing further invasions to simulate the opening up of a new pathway, SOM still performed with a high degree of accuracy.

The results presented here along with those presented by [14], which showed significant resilience to errors in presence/absence data, indicate the effectiveness of this tool in ranking potential invasive species. In addition, the number of species that can be simultaneously analysed using this technique is at present only limited by the availability of data and the amount of virtual computer memory available on a desktop. At present, on a 32 bit architecture desktop computer, we have been able to analyse data sets with up to 10,000 species (unpublished data).

A SOM analysis could be utilised by government agencies concerned with prioritising across large numbers of potentially invasive species in two ways. Firstly, a SOM could be used as an initial screening process to reduce the number of potential invasives to a more manageable number. Secondly, the species likelihood indices generated by a SOM analysis could be included in the consultative process by providing a ‘second opinion’ for both clarification and revision of expert opinion [14]; a critical, but often ignored part of the elicitation process [11]. In addition, these quantitative estimates of establishment could also be incorporated into economic models used in import risk assessments [16], [17] or by border biosecurity officers wanting to judge the likelihood of establishment for a recently intercepted invasive species.

While this work focuses on invasive species distributions, SOM could also be used to rank native species vulnerability. The SOM estimates a species' strength of association with an assemblage in a particular location, and this could therefore be used as a measure of a native species' strength of association, which would be a measure of its vulnerability. In the same way that an invasive pest assemblage captures the ecological, biological, and abiotic characteristics of a region, the native species assemblage would do the same. As such, by clustering regions, or more likely grid cells, a SOM could estimate a species' strength of association with a particular grid cell. In contrast to a SOM analysis of invasive species, which can highlight species absent from a location with high likelihood values, a SOM analysis of native species will highlight species present in a location with low likelihood values. It is these native species which will have a low strength of association with the species assemblage of a particular grid cell and, even though they are present in that location may have significantly reduced likelihood of persistence, relative to the other species present. Values across a native species' entire range could be combined to generate an overall metric for vulnerability. Generating such a metric for all native species in a dataset would enable ranking of native species vulnerabilities, which could be utilised by policy officers required to prioritise conservation efforts.

Overall, the results presented here provide further evidence of the power and reliability of a SOM analysis in predicting and ranking invasive species and we encourage its adoption by researchers and stakeholders.

## Materials and Methods

### Fungal pathogen dataset and SOM analysis

Fungal pathogen distribution data detailing the presence/absence data for 486 fungal pathogen species of plants over 420 regions of the world were extracted from the CABI Crop Protection Compendium [18]. This compendium is a database compiling information on all aspects of plant health and the distributional data are sourced from available literature records (http://www.cabi.org/cpc/default.aspx?site=161&page=1385). There are 459 regions defined by this compendium, which are political countries with many of the larger countries further subdivided into states or provinces (e.g. USA, China, Canada, Australia). Of the 459 regions, 39 regions had no fungal pathogens present and were removed from the analysis.

A 420×486 matrix was subsequently generated in which the presence or absence of each species was recorded in each region. The number of neurons in a SOM is partially determined by the heuristic rule suggested by [19], which is 5√n, where n is the number of samples. In addition, the two largest eigenvalues are calculated from the data set and the ratio of the length and width of the SOM is set to those eigenvalues. Given this ratio, the final number of neurons is set as close to Vesanto's heuristic rule as possible. The dimensions of the SOM used in this analysis was 13×8 (104 neurons) with the standard hexagonal lattice configuration and number of iterations: 52,000 [20].

The final neuron weight vector was comprised of 486 elements with each element representing each of the 486 species and having a value between 0 and 1. Each element can be interpreted as a likelihood index, or an index of how strongly that species is associated with other species in that neuron, and hence the species assemblage of any region associated with that neuron. This value can then be used to rank all species in a region from highest to lowest likelihood. It should be noted then that the SOM generates a likelihood index for all species, whether that species is present or absent in that region. A full explanation of a SOM analysis can be obtained from [20], [21].

The SOM analysis was performed on the fungal pathogen data and a likelihood of establishment list (top 100) generated for Australia and its states and territories.

### Virtual World

To test the reliability of these lists of fungal pathogens for Australia, we created a simulated “virtual world” within which the potential distribution patterns of pathogens are known. An essential property of this artificial world is that realistic species associations are present, providing the basis for the SOM analysis. Indices were assigned to each region to represent an invasibility index (InI) and to the pathogens to represent their invasion requirements (IRI) in terms of the regional susceptibility categories. We used these categories as the factor(s) that facilitate invasion likelihood are often poorly characterised [22], [23].

**Figure 6:**
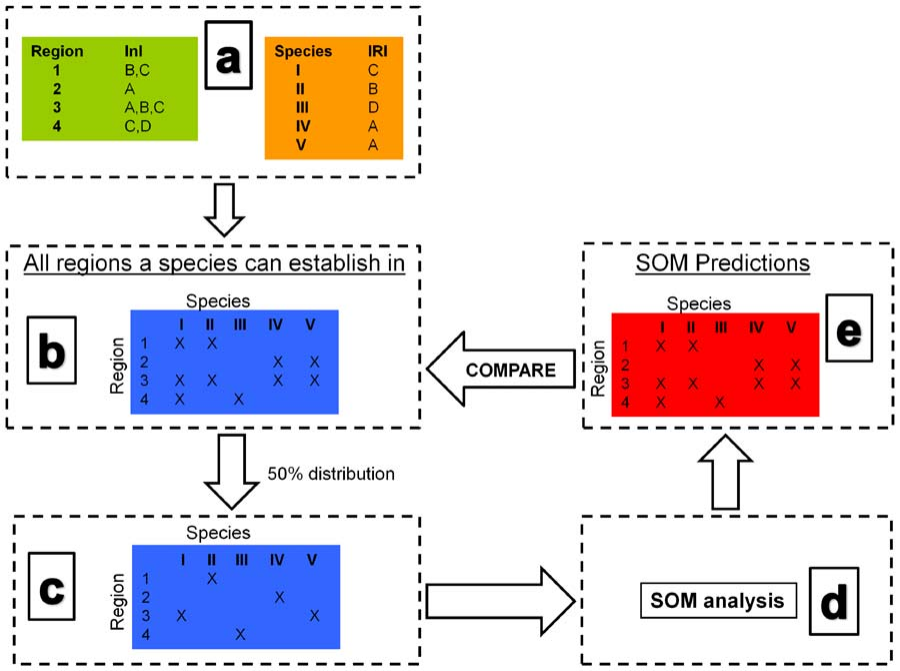
Virtual World Setup. Pictorial representation of how the virtual world was established and used to test SOM's ability to predict invasive species. (a) Characteristics assigned to regions and species (InI = invasibility index; IRI = invasion requirement index), (b) These characteristics used to determine which species can establish in which regions, (c) Species subsequently distributed to a predetermined proportion of the regions they could establish in (e.g. 50%), (d) This information analysed by SOM, and (e) SOM's predictions compared to the ‘truth’ (b).

An arbitrary ten categories of InI (A–J) were defined, which may be interpreted as the set of characteristics present in a region, which determine if a pathogen can establish. Adjacent categories (e.g. A and B) were considered more similar than non-adjacent categories (e.g. A and J). Each region could contain more than one category, but only adjacent or sequential categories (Figure 6a). For example, a region with three categories could have A, B, C, or B,C, D, or C,D, E, etc, but could not have A, D, J, or B, C, H. If a region has only a single InI (e.g. C), there is only a limited suite of species that could establish in this region. In contrast, if a region had all ten InI's (A–J), all species could establish.

Fungal pathogen species were randomly allocated only one of the ten possible IRI's (A–J) (Figure 6a). This index can be thought of as the characteristics of an invasive species that enables it to establish in a region. If a species' IRI matched a region's InI, then that species was able to establish (Figure 6b).

By creating a virtual world in this way we were able to create species associations, which are the patterns that the SOM looks for in its predictions. For example, all species with the same IRI are able to establish in exactly the same regions and would have a strong species association. If two species had IRI's that were adjacent to each other (e.g. A and B), they would often be able to establish in the same regions, but not as often as species that had the same IRI (i.e. they could both establish only in regions whose IRI spanned A and B). Finally, if two species had IRI's that were not ‘close’ to each other (e.g. A and J), they would not have a strong association. Only regions containing all ten InI's (A to J) would be susceptible to invasion from these two species.

We aimed to create a virtual world in which InI ranges reflected that of the real world. However, the potential distributions of invasive species across the real world's regions are unknown as species have not invaded all possible regions. We therefore used a proxy to determine the InI distribution by examining the worldwide plant species diversity [24], assuming diversity of native species reflects the final diversity of invasive species at large scales [25], [26]. Using this dataset, we determined the species richness distribution across the real world's regions, whereby the maximum species richness recorded was 10,000 species. We scaled the InI distributions of our virtual world to the distribution of plant species richness across regions of the real world (Table 3). For instance, 52.7% of the regions of the real world contained 1 to 1000 plant species, thus each region in the virtual region had a probability of 0.527 of receiving only one InI. Once it was determined that a region would receive only one InI, the InI was randomly selected from the ten possibilities (A to J), with each having an equal likelihood of being selected. Further, each region had a probability of 0.210 of receiving two InIs. There were nine possible pairings of InIs (AB, BC, CD, DE, EF, FG, GH, HI, IJ) as only adjacent InIs can be paired. If a region was selected to receive two InIs, one of the nine pairings was selected randomly, with each pairing having an equal likelihood of selection.

**Table 3:** Weighting for the random selection of invasibility indices (InI) for regions in the virtual world.

# species	range^1^	# ecoregions	proportion^2^
1–1,000	1	456	0.527
1,001–2,000	2	182	0.210
2,001–3,000	3	111	0.128
3,001–4,000	4	52	0.060
4,001–5,000	5	24	0.028
5,001–6,000	6	14	0.016
6,001–7,000	7	14	0.016
7,001–8,000	8	6	0.007
8,001–9,000	9	5	0.006
9,001–10,000	10	1	0.001

The number of ecoregions falling into each species range category was derived from native plant distributions [24].

1 The number of InI's given to a region in the virtual world.

2 The proportion of all ecoregions falling into the species category.

Once the invasive species' IRI and the regions' InI are allocated, the potential distribution of species across regions is known (the ‘fully invaded’ distribution) (Figure 6b). The current real world distribution of invasive pathogens represents some unknown point before this fully invaded distribution. To validate SOM predictions, we allocated each species to a pre-determined percentage of all possible regions it could invade (Figure 6c). For example, if a species was able to invade 200 of the 420 regions in the virtual world, we could randomly allocate that species to 50% of those 200 regions. These restricted species distributions could then be analysed using SOM (Figure 6d) and its predictions compared to the known fully invaded distribution (Figure 6e).

### Scenarios

We generated 200 virtual worlds in which each region's InI and species' IRI were randomly selected. Each virtual world was therefore unique. For 100 virtual worlds we allocated species to 50% of their potential range (scenario 1) and for the remaining 100 virtual worlds we allocated species to 20% of their potential range (scenario 2). Distributing species to either 20% or 50% of their potential range gave the SOM different levels of information on potential species distributions, and represents a conservative and more realistic scenario, respectively. As with the fungal pathogen dataset, each dataset was a matrix (420×486) in which a row represented a region and a column represented a species. The matrix therefore contained 1's and 0's representing the presence or absence of all species in all regions. Each dataset was then analysed using SOM and ranked species lists generated for each region in each virtual world.

We determined which scenario (20% or 50%) was most similar to the fungal pathogen dataset. We combined all 100 virtual worlds from each scenario, which created a negative binomial-distributed data set for each scenario containing the relative frequency of each number of species in a region. We then calculated maximum likelihood estimates for the mean and dispersion parameters for each negative binomial distribution [27]. We subsequently calculated the log-likelihood of collecting the original fungal pathogen data set, given that the true fungal pathogen distribution is equal to the negative binomial distribution generated by each scenario (20% or 50%). We calculated an AIC value from this log-likelihood value (with two parameters for the negative binomial distribution) for each scenario, to compare the abilities of the two scenarios to explain the data, and the lowest AIC value indicated which scenario was most similar to the fungal pathogen dataset.

The SOM analysis was performed using Matlab [28] and the SOM Toolbox (version 2.0) developed by the Laboratory of Information and Computer Science Helsinki University of Technology (http://www.cis.hut.fi/projects/somtoolbox/), while the AIC test used R [29].

### Assessing SOM

#### Regional success rate

The ranked list for each region generated by SOM was used to evaluate the SOM performance (species were ranked from highest likelihood of establishing to lowest). For every region, we determined how many absent species could establish and calculated the proportion of absent species that were ranked in that top part of the list. For example, if there were 152 absent species that could establish in a region and 147 of those species were ranked in the top 152 of the list, then the regional success rate for that region would be 0.97 (97%). For convenience, the top 152 species in this list is referred to as the ‘top half’ and the remainder is referred to as the ‘bottom half’. It should be noted that the size of the ‘top half’ and ‘bottom half’ is determined by the number of species that can establish in that region and will vary between regions.

For each virtual world there were 420 regions. Within each scenario, all the regional success rates in all the virtual worlds were combined (42,000 regions) to give an overall mean success rate across all virtual worlds in each scenario.

#### Species success rate

We also calculated the success rate for each species across all regions. If a species could establish in a region, we counted a success if that species was correctly ranked in the ‘top half’ of that region's list. If a species could not establish in a region then we counted a success if that species was correctly ranked in the ‘bottom half’ of that region's list.

For each virtual world there were 486 species. Within each scenario, all the species success rates in all the virtual worlds, were combined (48,600 species) to give an overall mean success rate across all virtual worlds in each scenario.

## Supporting Information

Table S1 The top 100 list for plant pathogen species absent from Australia.
(DOC)

Table S2 The top 100 list for plant pathogen species absent from Western Australia.
(DOC)

Table S3 The top 100 list for plant pathogen species absent from South Australia.
(DOC)

Table S4 The top 100 list for plant pathogen species absent from Victoria.
(DOC)

Table S5 The top 100 list for plant pathogen species absent from Queensland.
(DOC)

Table S6 The top 100 list for plant pathogen species absent from New South Wales.
(DOC)

Table S7 The top 100 list for plant pathogen species absent from the Northern Territory.
(DOC)

Table S8 The top 100 list for plant pathogen species absent from Tasmania.
(DOC)

Table S9 Full list of neurons and associated regions from the SOM analysis of fungal pathogen assemblages.
(DOC)

Table S10 Acceptability of lists generated for regions with less than 8 species present.
(DOC)
