# Supplementary material for: Predicting Invasive Fungal Pathogens Using Invasive Pest Assemblages: Testing Model Predictions in a Virtual World
Source: PLoS One. 2011 Oct 10;6(10):e25695. doi: 10.1371/journal.pone.0025695 (PMC3189937; doi:10.1371/journal.pone.0025695)
Supplement: Table S1 — The top 100 list for plant pathogen species absent from Australia. (DOC) [file pone.0025695.s001.doc]

Table S1. The top 100 list for plant pathogen species absent from Australia.

| **Rank** | **Species Name** | **Likelihood Index** | **Rank** | **Species Name** | **Likelihood Index** | **Rank** | **Species Name** | **Likelihood Index** |
| --- | --- | --- | --- | --- | --- | --- | --- | --- |
| 1 | *Ustilago avenae* | 0.9107 | 35 | *Alternaria japonica* | 0.517 | 69 | *Mycosphaerella cruenta* | 0.3162 |
| 2 | *Phytophthora infestans* | 0.8872 | 36 | *Fusarium oxysporum f.sp. lini* | 0.5161 | 70 | *Phytophthora colocasiae* | 0.3157 |
| 3 | *Mycovellosiella fulva* | 0.8867 | 37 | *Pyrenophora tritici-repentis* | 0.5118 | 71 | *Verticillium albo-atrum* | 0.309 |
| 4 | *Cochliobolus sativus* | 0.8656 | 38 | *Nectria galligena* | 0.492 | 72 | *Ulocladium atrum* | 0.3072 |
| 5 | *Setosphaeria turcica* | 0.812 | 39 | *Cronartium ribicola* | 0.4839 | 73 | *Asperisporium caricae* | 0.307 |
| 6 | *Phytophthora nicotianae* | 0.8069 | 40 | *Fusarium sacchari* | 0.4712 | 74 | *Armillaria ostoyae* | 0.303 |
| 7 | *Sporisorium sorghi* | 0.8067 | 41 | *Ceratocystis fimbriata* | 0.4634 | 75 | *Monosporascus cannonballus* | 0.303 |
| 8 | *Gaeumannomyces graminis var. tritici* | 0.789 | 42 | *Septoria cannabis* | 0.4511 | 76 | *Ophiostoma piceae* | 0.3022 |
| 9 | *Claviceps purpurea* | 0.7766 | 43 | *Pythium splendens* | 0.4477 | 77 | *Pseudoperonospora cannabina* | 0.3021 |
| 10 | *Puccinia striiformis* | 0.7655 | 44 | *Cryphonectria parasitica* | 0.4405 | 78 | *Cronartium quercuum* | 0.2935 |
| 11 | *Phaeosphaeria nodorum* | 0.7487 | 45 | *Didymella lycopersici* | 0.4314 | 79 | *Aecidium mori* | 0.2931 |
| 12 | *Colletotrichum orbiculare* | 0.7476 | 46 | *Podosphaera macularis* | 0.43 | 80 | *Olpidium brassicae* | 0.2918 |
| 13 | *Mycosphaerella graminicola* | 0.729 | 47 | *Passalora sojina* | 0.4287 | 81 | *Uredo cajani* | 0.2871 |
| 14 | *Sclerotinia sclerotiorum* | 0.7131 | 48 | *Cercospora sorghi* | 0.4226 | 82 | *Mycosphaerella pruni-persicae* | 0.2857 |
| 15 | *Sporisorium cruentum* | 0.7105 | 49 | *Nematospora coryli* | 0.4189 | 83 | *Alternaria solani* | 0.2825 |
| 16 | *Rosellinia necatrix* | 0.7089 | 50 | *Didymella rabiei* | 0.4176 | 84 | *Ustilago shiraiana* | 0.2794 |
| 17 | *Septoria apiicola* | 0.6834 | 51 | *Mycosphaerella pyri* | 0.4164 | 85 | *Gymnosporangium fuscum* | 0.2761 |
| 18 | *Cladosporium cucumerinum* | 0.6832 | 52 | *Phomopsis asparagi* | 0.4025 | 86 | *Fusarium oxysporum f.sp. lentis* | 0.2724 |
| 19 | *Phytophthora capsici* | 0.6762 | 53 | *Erysiphe orontii* | 0.3991 | 87 | *Stagonospora sacchari* | 0.2582 |
| 20 | *Sclerospora graminicola* | 0.6736 | 54 | *Alternaria sesami* | 0.3989 | 88 | *Cryptodiaporthe populea* | 0.2552 |
| 21 | *Botrytis fabae* | 0.6662 | 55 | *Fomes fomentarius* | 0.3914 | 89 | *Lachnella willkommii* | 0.2512 |
| 22 | *Puccinia graminis* | 0.6454 | 56 | *Colletotrichum linicola* | 0.3803 | 90 | *Monographella nivalis* | 0.2476 |
| 23 | *Colletotrichum sublineolum* | 0.6375 | 57 | *Septoria glycines* | 0.3766 | 91 | *Phialophora cinerescens* | 0.2474 |
| 24 | *Fusarium oxysporum f.sp. vasinfectum* | 0.6291 | 58 | *Mycosphaerella gibsonii* | 0.3621 | 92 | *Peronosclerospora philippinensis* | 0.2464 |
| 25 | *Puccinia asparagi* | 0.5987 | 59 | *Hemileia vastatrix* | 0.3565 | 93 | *Synchytrium endobioticum* | 0.2392 |
| 26 | *Leptosphaeria coniothyrium* | 0.597 | 60 | *Guignardia bidwellii* | 0.3508 | 94 | *Hypocrea rufa* | 0.2386 |
| 27 | *Phaeolus schweinitzii* | 0.5839 | 61 | *Didymella lentis* | 0.3447 | 95 | *Armillaria mellea* | 0.237 |
| 28 | *Bipolaris sacchari* | 0.5801 | 62 | *Kabatiella zeae* | 0.3399 | 96 | *Moesziomyces bullatus* | 0.2334 |
| 29 | *Puccinia coronata* | 0.5606 | 63 | *Cronartium flaccidum* | 0.3383 | 97 | *Exobasidium vexans* | 0.2329 |
| 30 | *Colletotrichum circinans* | 0.5552 | 64 | *Aphanomyces euteiches* | 0.3359 | 98 | *Fomitopsis pinicola* | 0.2327 |
| 31 | *Monilinia fructigena* | 0.5497 | 65 | *Ceratocystis ulmi* | 0.3311 | 99 | *Fusarium oxysporum f.sp. batatas* | 0.2299 |
| 32 | *Puccinia purpurea* | 0.5405 | 66 | *Peronospora viciae* | 0.3277 | 100 | *Corticium koleroga* | 0.2296 |
| 33 | *Ustilago crameri* | 0.5273 | 67 | *Colletotrichum tabacum* | 0.3272 |  |  |  |
| 34 | *Pseudoperonospora humuli* | 0.5223 | 68 | *Elsino veneta* | 0.3211 |  |  |  |
