# Supplementary material for: Predicting Invasive Fungal Pathogens Using Invasive Pest Assemblages: Testing Model Predictions in a Virtual World
Source: PLoS One. 2011 Oct 10;6(10):e25695. doi: 10.1371/journal.pone.0025695 (PMC3189937; doi:10.1371/journal.pone.0025695)
Supplement: Table S3 — The top 100 list for plant pathogen species absent from South Australia. (DOC) [file pone.0025695.s003.doc]

Table S3. The top 100 list for plant pathogen species absent from South Australia.

| **Rank** | **Species Name** | **Likelihood Index** | **Rank** | **Species Name** | **Likelihood Index** | **Rank** | **Species Name** | **Likelihood Index** |
| --- | --- | --- | --- | --- | --- | --- | --- | --- |
| 1 | *Puccinia triticina* | 0.6744 | 35 | *Cochliobolus miyabeanus* | 0.3509 | 69 | *Ceratobasidium cereale* | 0.2421 |
| 2 | *Ustilago zeae* | 0.6740 | 36 | *Rosellinia necatrix* | 0.3505 | 70 | *Alternaria longipes* | 0.2412 |
| 3 | *Puccinia allii* | 0.6304 | 37 | *Ulocladium atrum* | 0.3472 | 71 | *Ustilago crameri* | 0.2402 |
| 4 | *Sclerotinia sclerotiorum* | 0.6194 | 38 | *Puccinia purpurea* | 0.3419 | 72 | *Trichoderma harzianum* | 0.2378 |
| 5 | *Leveillula taurica* | 0.5859 | 39 | *Mycosphaerella pini* | 0.3396 | 73 | *Colletotrichum truncatum* | 0.2373 |
| 6 | *Peronospora destructor* | 0.5847 | 40 | *Blumeria graminis* | 0.3308 | 74 | *Botrytis fabae* | 0.2353 |
| 7 | *Sphacelotheca reiliana* | 0.5552 | 41 | *Fusarium oxysporum f.sp. vasinfectum* | 0.3282 | 75 | *Ustilago scitaminea* | 0.2336 |
| 8 | *Gibberella avenacea* | 0.5498 | 42 | *Discosphaerina fulvida* | 0.3276 | 76 | *Phragmidium rubi-idaei* | 0.2331 |
| 9 | *Alternaria brassicicola* | 0.5464 | 43 | *Podosphaera macularis* | 0.3255 | 77 | *Spilocaea oleaginea* | 0.2283 |
| 10 | *Pleospora betae* | 0.5365 | 44 | *Fusarium oxysporum f.sp. lini* | 0.3164 | 78 | *Phaeosphaeria avenaria f.sp. avenaria* | 0.2242 |
| 11 | *Setosphaeria turcica* | 0.5327 | 45 | *Guignardia bidwellii* | 0.3161 | 79 | *Verticillium albo-atrum* | 0.2234 |
| 12 | *Monilinia fructigena* | 0.5192 | 46 | *Stemphylium sarciniforme* | 0.3146 | 80 | *Cochliobolus heterostrophus* | 0.2213 |
| 13 | *Venturia inaequalis* | 0.5077 | 47 | *Phytophthora capsici* | 0.3122 | 81 | *Aspergillus niger* | 0.2210 |
| 14 | *Pyrenophora tritici-repentis* | 0.5070 | 48 | *Cronartium ribicola* | 0.3089 | 82 | *Nattrassia mangiferae* | 0.2188 |
| 15 | *Pyrenophora graminea* | 0.5063 | 49 | *Didymella rabiei* | 0.3086 | 83 | *Alternaria dianthicola* | 0.2186 |
| 16 | *Puccinia sorghi* | 0.5022 | 50 | *Didymella lycopersici* | 0.3065 | 84 | *Pleiochaeta setosa* | 0.2163 |
| 17 | *Peronospora farinosa* | 0.4907 | 51 | *Colletotrichum acutatum* | 0.3029 | 85 | *Chrysomyxa abietis* | 0.2137 |
| 18 | *Sclerotinia trifoliorum* | 0.4797 | 52 | *Phaeolus schweinitzii* | 0.2833 | 86 | *Pezicula malicorticis* | 0.2136 |
| 19 | *Albugo candida* | 0.4486 | 53 | *Alternaria citri* | 0.2832 | 87 | *Botryosphaeria ribis* | 0.2113 |
| 20 | *Nectria galligena* | 0.4444 | 54 | *Helminthosporium solani* | 0.2827 | 88 | *Entyloma dahliae* | 0.2101 |
| 21 | *Magnaporthe grisea* | 0.4281 | 55 | *Ceratocystis ulmi* | 0.2813 | 89 | *Mycosphaerella pyri* | 0.2062 |
| 22 | *Pseudoperonospora humuli* | 0.4226 | 56 | *Cronartium flaccidum* | 0.2805 | 90 | *Blumeriella jaapii* | 0.2044 |
| 23 | *Monographella nivalis* | 0.4195 | 57 | *Gymnosporangium fuscum* | 0.2790 | 91 | *Peronospora viciae* | 0.2015 |
| 24 | *Podosphaera pannosa* | 0.4171 | 58 | *Fusarium oxysporum f.sp. melonis* | 0.2687 | 92 | *Elsino? fawcettii* | 0.1979 |
| 25 | *Podosphaera aphanis* | 0.4143 | 59 | *Sporisorium cruentum* | 0.2655 | 93 | *Glomerella tucumanensis* | 0.1930 |
| 26 | *Spilocaea pyracanthae* | 0.4116 | 60 | *Peronospora manshurica* | 0.2622 | 94 | *Ceratocystis paradoxa* | 0.1928 |
| 27 | *Cladosporium cucumerinum* | 0.3946 | 61 | *Colletotrichum linicola* | 0.2591 | 95 | *Ramularia onobrychidis* | 0.1927 |
| 28 | *Didymella applanata* | 0.3945 | 62 | *Coniella diplodiella* | 0.2556 | 96 | *Sclerospora graminicola* | 0.1895 |
| 29 | *Fusarium culmorum* | 0.3807 | 63 | *Mycosphaerella rabiei* | 0.2548 | 97 | *Cryphonectria parasitica* | 0.1895 |
| 30 | *Magnaporthe salvinii* | 0.3673 | 64 | *Glomerella cingulata* | 0.2517 | 98 | *Phaeocryptopus gaeumannii* | 0.1829 |
| 31 | *Puccinia asparagi* | 0.3587 | 65 | *Mycosphaerella berkeleyi* | 0.2470 | 99 | *Phaeoisariopsis griseola* | 0.1795 |
| 32 | *Heterobasidion annosum* | 0.3550 | 66 | *Septoria cannabis* | 0.2446 | 100 | *Botrytis anthophila* | 0.1786 |
| 33 | *Helicobasidium brebissonii* | 0.3542 | 67 | *Fusarium oxysporum f.sp. niveum* | 0.2431 |  |  |  |
| 34 | *Uromyces ciceris-arietini* | 0.3519 | 68 | *Venturia cerasi* | 0.2421 |  |  |  |
