# Supplementary material for: Predicting Invasive Fungal Pathogens Using Invasive Pest Assemblages: Testing Model Predictions in a Virtual World
Source: PLoS One. 2011 Oct 10;6(10):e25695. doi: 10.1371/journal.pone.0025695 (PMC3189937; doi:10.1371/journal.pone.0025695)
Supplement: Table S4 — The top 100 list for plant pathogen species absent from Victoria. (DOC) [file pone.0025695.s004.doc]

Table S4. The top 100 list for plant pathogen species absent from Victoria.

| **Rank** | **Species Name** | **Likelihood Index** | **Rank** | **Species Name** | **Likelihood Index** | **Rank** | **Species Name** | **Likelihood Index** |
| --- | --- | --- | --- | --- | --- | --- | --- | --- |
| 1 | *Ustilago hordei* | 0.8612 | 35 | *Diplocarpon earlianum* | 0.3570 | 69 | *Drepanopeziza ribis* | 0.2630 |
| 2 | *Puccinia triticina* | 0.6744 | 36 | *Pyrenophora chaetomioides* | 0.3550 | 70 | *Peronospora manshurica* | 0.2622 |
| 3 | *Ustilago zeae* | 0.6740 | 37 | *Heterobasidion annosum* | 0.3550 | 71 | *Colletotrichum linicola* | 0.2591 |
| 4 | *Puccinia allii* | 0.6304 | 38 | *Plasmopara halstedii* | 0.3545 | 72 | *Coniella diplodiella* | 0.2556 |
| 5 | *Rhynchosporium secalis* | 0.6185 | 39 | *Helicobasidium brebissonii* | 0.3542 | 73 | *Mycosphaerella rabiei* | 0.2548 |
| 6 | *Macrophomina phaseolina* | 0.6171 | 40 | *Uromyces ciceris-arietini* | 0.3519 | 74 | *Myrothecium roridum* | 0.2539 |
| 7 | *Puccinia coronata* | 0.6122 | 41 | *Cochliobolus miyabeanus* | 0.3509 | 75 | *Glomerella cingulata* | 0.2517 |
| 8 | *Peronospora destructor* | 0.5847 | 42 | *Rosellinia necatrix* | 0.3505 | 76 | *Mycosphaerella berkeleyi* | 0.2470 |
| 9 | *Claviceps purpurea* | 0.5608 | 43 | *Puccinia purpurea* | 0.3419 | 77 | *Penicillium digitatum* | 0.2457 |
| 10 | *Pyrenophora teres* | 0.5333 | 44 | *Fusarium oxysporum f.sp. cucumerinum* | 0.3321 | 78 | *Septoria cannabis* | 0.2446 |
| 11 | *Monilinia fructigena* | 0.5192 | 45 | *Blumeria graminis* | 0.3308 | 79 | *Fusarium oxysporum f.sp. niveum* | 0.2431 |
| 12 | *Venturia inaequalis* | 0.5077 | 46 | *Colletotrichum orbiculare* | 0.3287 | 80 | *Venturia cerasi* | 0.2421 |
| 13 | *Pyrenophora graminea* | 0.5063 | 47 | *Fusarium oxysporum f.sp. vasinfectum* | 0.3282 | 81 | *Ceratobasidium cereale* | 0.2421 |
| 14 | *Puccinia sorghi* | 0.5022 | 48 | *Tilletia controversa* | 0.3265 | 82 | *Alternaria longipes* | 0.2412 |
| 15 | *Peronospora farinosa* | 0.4907 | 49 | *Podosphaera macularis* | 0.3255 | 83 | *Ustilago crameri* | 0.2402 |
| 16 | *Chalara elegans* | 0.4805 | 50 | *Fusarium oxysporum f.sp. lini* | 0.3164 | 84 | *Colletotrichum truncatum* | 0.2373 |
| 17 | *Botrytis tulipae* | 0.4593 | 51 | *Guignardia bidwellii* | 0.3161 | 85 | *Botrytis fabae* | 0.2353 |
| 18 | *Albugo candida* | 0.4486 | 52 | *Stemphylium sarciniforme* | 0.3146 | 86 | *Puccinia hordei* | 0.2341 |
| 19 | *Nectria galligena* | 0.4444 | 53 | *Phytophthora capsici* | 0.3122 | 87 | *Ustilago scitaminea* | 0.2336 |
| 20 | *Phoma medicaginis var. medicaginis* | 0.4404 | 54 | *Cronartium ribicola* | 0.3089 | 88 | *Spilocaea oleaginea* | 0.2283 |
| 21 | *Leptosphaeria coniothyrium* | 0.4382 | 55 | *Didymella rabiei* | 0.3086 | 89 | *Phaeosphaeria avenaria f.sp. avenaria* | 0.2242 |
| 22 | *Magnaporthe grisea* | 0.4281 | 56 | *Didymella lycopersici* | 0.3065 | 90 | *Cochliobolus heterostrophus* | 0.2213 |
| 23 | *Pseudoperonospora humuli* | 0.4226 | 57 | *Phytophthora fragariae* | 0.2967 | 91 | *Aspergillus niger* | 0.2210 |
| 24 | *Podosphaera pannosa* | 0.4171 | 58 | *Phaeolus schweinitzii* | 0.2833 | 92 | *Nattrassia mangiferae* | 0.2188 |
| 25 | *Podosphaera aphanis* | 0.4143 | 59 | *Alternaria citri* | 0.2832 | 93 | *Alternaria dianthicola* | 0.2186 |
| 26 | *Spilocaea pyracanthae* | 0.4116 | 60 | *Ceratocystis ulmi* | 0.2813 | 94 | *Pleiochaeta setosa* | 0.2163 |
| 27 | *Urocystis cepulae* | 0.4114 | 61 | *Cronartium flaccidum* | 0.2805 | 95 | *Chrysomyxa abietis* | 0.2137 |
| 28 | *Erysiphe necator* | 0.3957 | 62 | *Gymnosporangium fuscum* | 0.2790 | 96 | *Pezicula malicorticis* | 0.2136 |
| 29 | *Cladosporium cucumerinum* | 0.3946 | 63 | *Kabatiella caulivora* | 0.2765 | 97 | *Botryosphaeria ribis* | 0.2113 |
| 30 | *Lophodermium pinastri* | 0.3812 | 64 | *Penicillium italicum* | 0.2730 | 98 | *Entyloma dahliae* | 0.2101 |
| 31 | *Puccinia carthami* | 0.3812 | 65 | *Sclerophthora macrospora* | 0.2721 | 99 | *Mycosphaerella pyri* | 0.2062 |
| 32 | *Magnaporthe salvinii* | 0.3673 | 66 | *Colletotrichum circinans* | 0.2707 | 100 | *Blumeriella jaapii* | 0.2044 |
| 33 | *Puccinia pelargonii-zonalis* | 0.3590 | 67 | *Sporisorium cruentum* | 0.2655 |  |  |  |
| 34 | *Puccinia asparagi* | 0.3587 | 68 | *Physoderma alfalfae* | 0.2645 |  |  |  |
