# Supplementary material for: Predicting Invasive Fungal Pathogens Using Invasive Pest Assemblages: Testing Model Predictions in a Virtual World
Source: PLoS One. 2011 Oct 10;6(10):e25695. doi: 10.1371/journal.pone.0025695 (PMC3189937; doi:10.1371/journal.pone.0025695)
Supplement: Table S5 — The top 100 list for plant pathogen species absent from Queensland. (DOC) [file pone.0025695.s005.doc]

Table S5. The top 100 list for plant pathogen species absent from Queensland.

| **Rank** | **Species Name** | **Likelihood Index** | **Rank** | **Species Name** | **Likelihood Index** | **Rank** | **Species Name** | **Likelihood Index** |
| --- | --- | --- | --- | --- | --- | --- | --- | --- |
| 1 | *Ustilago scitaminea* | 0.8770 | 35 | *Botrytis aclada* | 0.4905 | 69 | *Mycena citricolor* | 0.3215 |
| 2 | *Cercospora beticola* | 0.8619 | 36 | *Phytophthora capsici* | 0.4830 | 70 | *Phomopsis theae* | 0.3209 |
| 3 | *Puccinia sorghi* | 0.8144 | 37 | *Rhynchosporium secalis* | 0.4747 | 71 | *Didymella lycopersici* | 0.3167 |
| 4 | *Puccinia triticina* | 0.7973 | 38 | *Sarocladium oryzae* | 0.4699 | 72 | *Trichoderma harzianum* | 0.3143 |
| 5 | *Cochliobolus miyabeanus* | 0.7940 | 39 | *Cochliobolus lunatus* | 0.4667 | 73 | *Colletotrichum kahawae* | 0.3123 |
| 6 | *Colletotrichum gossypii* | 0.7833 | 40 | *Phytophthora cactorum* | 0.4595 | 74 | *Guignardia bidwellii* | 0.3109 |
| 7 | *Puccinia striiformis* | 0.7786 | 41 | *Monographella albescens* | 0.4567 | 75 | *Alternaria japonica* | 0.3099 |
| 8 | *Lasiodiplodia theobromae* | 0.7562 | 42 | *Puccinia asparagi* | 0.4480 | 76 | *Urocystis cepulae* | 0.3069 |
| 9 | *Alternaria longipes* | 0.7546 | 43 | *Plasmopara halstedii* | 0.4439 | 77 | *Alternaria alternata* | 0.3067 |
| 10 | *Hemileia vastatrix* | 0.7450 | 44 | *Rosellinia necatrix* | 0.4427 | 78 | *Puccinia psidii* | 0.3048 |
| 11 | *Sphaerulina oryzina* | 0.7320 | 45 | *Entyloma oryzae* | 0.4412 | 79 | *Blumeria graminis* | 0.2984 |
| 12 | *Ustilaginoidea virens* | 0.7207 | 46 | *Helminthosporium solani* | 0.4387 | 80 | *Uromyces ciceris-arietini* | 0.2973 |
| 13 | *Myrothecium roridum* | 0.7184 | 47 | *Sphaceloma perseae* | 0.4343 | 81 | *Cryphonectria cubensis* | 0.2961 |
| 14 | *Botryosphaeria ribis* | 0.7037 | 48 | *Diaporthe phaseolorum var. sojae* | 0.4274 | 82 | *Phomopsis asparagi* | 0.2937 |
| 15 | *Pythium aphanidermatum* | 0.6959 | 49 | *Pyrenophora graminea* | 0.4221 | 83 | *Phaeolus schweinitzii* | 0.2934 |
| 16 | *Sporisorium cruentum* | 0.6955 | 50 | *Mycosphaerella gibsonii* | 0.4152 | 84 | *Armillaria heimii* | 0.2917 |
| 17 | *Asperisporium caricae* | 0.6479 | 51 | *Coleosporium ipomoeae* | 0.4149 | 85 | *Septoria glycines* | 0.2889 |
| 18 | *Podosphaera pannosa* | 0.6396 | 52 | *Colletotrichum circinans* | 0.4114 | 86 | *Phaeoramularia angolensis* | 0.2795 |
| 19 | *Stenocarpella maydis* | 0.6381 | 53 | *Nectria rigidiuscula* | 0.4103 | 87 | *Puccinia carthami* | 0.2795 |
| 20 | *Albugo candida* | 0.6282 | 54 | *Pseudocercospora fuligena* | 0.4044 | 88 | *Camptomeris leucaenae* | 0.2793 |
| 21 | *Thanatephorus cucumeris* | 0.6237 | 55 | *Cladosporium cucumerinum* | 0.4040 | 89 | *Moesziomyces bullatus* | 0.2771 |
| 22 | *Gloeocercospora sorghi* | 0.6230 | 56 | *Lophodermium pinastri* | 0.3912 | 90 | *Alternaria padwickii* | 0.2740 |
| 23 | *Nematospora coryli* | 0.6204 | 57 | *Curvularia* | 0.3873 | 91 | *Uredo cajani* | 0.2726 |
| 24 | *Mycosphaerella henningsii* | 0.6193 | 58 | *Corticium koleroga* | 0.3833 | 92 | *Phakopsora meibomiae* | 0.2711 |
| 25 | *Puccinia allii* | 0.6151 | 59 | *Rigidoporus microporus* | 0.3777 | 93 | *Dactuliochaeta glycines* | 0.2703 |
| 26 | *Cercospora kikuchii* | 0.5905 | 60 | *Alternaria sesami* | 0.3768 | 94 | *Fusarium sporotrichioides* | 0.2664 |
| 27 | *Pyrenophora teres* | 0.5704 | 61 | *Rosellinia bunodes* | 0.3479 | 95 | *Gibberella avenacea* | 0.2614 |
| 28 | *Peronospora farinosa* | 0.5664 | 62 | *Phakopsora euvitis* | 0.3444 | 96 | *Microcyclus ulei* | 0.2540 |
| 29 | *Erysiphe necator* | 0.5513 | 63 | *Colletotrichum tabacum* | 0.3372 | 97 | *Gibberella xylarioides* | 0.2539 |
| 30 | *Cercospora zeae-maydis* | 0.5504 | 64 | *Stemphylium sarciniforme* | 0.3358 | 98 | *Claviceps fusiformis* | 0.2521 |
| 31 | *Passalora sojina* | 0.5480 | 65 | *Fusarium oxysporum f.sp. lini* | 0.3328 | 99 | *Colletotrichum capsici* | 0.2513 |
| 32 | *Aspergillus niger* | 0.5206 | 66 | *Lecanicillium lecanii* | 0.3321 | 100 | *Bipolaris heveae* | 0.2503 |
| 33 | *Venturia inaequalis* | 0.4999 | 67 | *Gibberella stilboides* | 0.3290 |  |  |  |
| 34 | *Sclerospora graminicola* | 0.4921 | 68 | *Cladosporium musae* | 0.3257 |  |  |  |
