# Supplementary material for: Predicting Invasive Fungal Pathogens Using Invasive Pest Assemblages: Testing Model Predictions in a Virtual World
Source: PLoS One. 2011 Oct 10;6(10):e25695. doi: 10.1371/journal.pone.0025695 (PMC3189937; doi:10.1371/journal.pone.0025695)
Supplement: Table S6 — The top 100 list for plant pathogen species absent from New South Wales. (DOC) [file pone.0025695.s006.doc]

Table S6. The top 100 list for plant pathogen species absent from New South Wales.

| **Rank** | **Species Name** | **Likelihood Index** | **Rank** | **Species Name** | **Likelihood Index** | **Rank** | **Species Name** | **Likelihood Index** |
| --- | --- | --- | --- | --- | --- | --- | --- | --- |
| 1 | *Puccinia triticina* | 0.9206 | 35 | *Colletotrichum gossypii* | 0.6049 | 69 | *Fusarium oxysporum f.sp. conglutinans* | 0.4387 |
| 2 | *Peronospora destructor* | 0.9136 | 36 | *Puccinia asparagi* | 0.5987 | 70 | *Nectria cinnabarina* | 0.4316 |
| 3 | *Cercospora beticola* | 0.9004 | 37 | *Ustilago scitaminea* | 0.5982 | 71 | *Didymella lycopersici* | 0.4314 |
| 4 | *Peronospora farinosa* | 0.8845 | 38 | *Phaeolus schweinitzii* | 0.5839 | 72 | *Podosphaera macularis* | 0.4301 |
| 5 | *Venturia inaequalis* | 0.8705 | 39 | *Ascochyta gossypii* | 0.5823 | 73 | *Passalora sojina* | 0.4287 |
| 6 | *Albugo candida* | 0.8554 | 40 | *Puccinia arachidis* | 0.5787 | 74 | *Ceratobasidium cereale* | 0.4283 |
| 7 | *Puccinia sorghi* | 0.8312 | 41 | *Fusarium oxysporum f.sp. niveum* | 0.5783 | 75 | *Botrytis tulipae* | 0.4273 |
| 8 | *Leveillula taurica* | 0.8079 | 42 | *Blumeria graminis* | 0.5771 | 76 | *Cercospora sorghi* | 0.4226 |
| 9 | *Magnaporthe grisea* | 0.7990 | 43 | *Fusarium oxysporum f.sp. cucumerinum* | 0.5674 | 77 | *Gloeocercospora sorghi* | 0.4221 |
| 10 | *Pyrenophora graminea* | 0.7904 | 44 | *Cochliobolus lunatus* | 0.5669 | 78 | *Nematospora coryli* | 0.4189 |
| 11 | *Botryosphaeria ribis* | 0.7418 | 45 | *Sphaerulina oryzina* | 0.5659 | 79 | *Paecilomyces lilacinus* | 0.4166 |
| 12 | *Alternaria alternata* | 0.7112 | 46 | *Pythium graminicola* | 0.5609 | 80 | *Mycosphaerella pyri* | 0.4164 |
| 13 | *Sporisorium cruentum* | 0.7105 | 47 | *Monilinia fructigena* | 0.5497 | 81 | *Podosphaera mors-uvae* | 0.4161 |
| 14 | *Rosellinia necatrix* | 0.7089 | 48 | *Didymella fabae* | 0.5460 | 82 | *Fusarium sporotrichioides* | 0.4142 |
| 15 | *Pythium aphanidermatum* | 0.6948 | 49 | *Gibberella intricans* | 0.5272 | 83 | *Fusarium oxysporum f.sp. melonis* | 0.4087 |
| 16 | *Lasiodiplodia theobromae* | 0.6871 | 50 | *Pseudoperonospora humuli* | 0.5223 | 84 | *Phomopsis asparagi* | 0.4025 |
| 17 | *Alternaria helianthi* | 0.6843 | 51 | *Alternaria japonica* | 0.5170 | 85 | *Erysiphe orontii* | 0.3991 |
| 18 | *Cladosporium cucumerinum* | 0.6832 | 52 | *Fusarium oxysporum f.sp. lini* | 0.5161 | 86 | *Alternaria sesami* | 0.3989 |
| 19 | *Phytophthora capsici* | 0.6762 | 53 | *Glomerella graminicola* | 0.5089 | 87 | *Fomes fomentarius* | 0.3914 |
| 20 | *Sclerospora graminicola* | 0.6736 | 54 | *Ustilaginoidea virens* | 0.5081 | 88 | *Monographella albescens* | 0.3897 |
| 21 | *Trichoderma harzianum* | 0.6725 | 55 | *Tilletia barclayana* | 0.5064 | 89 | *Alternaria dianthicola* | 0.3865 |
| 22 | *Podosphaera aphanis* | 0.6642 | 56 | *Nectria galligena* | 0.4920 | 90 | *Phytophthora medicaginis* | 0.3833 |
| 23 | *Lophodermium pinastri* | 0.6585 | 57 | *Mycosphaerella arachidis* | 0.4858 | 91 | *Colletotrichum linicola* | 0.3803 |
| 24 | *Colletotrichum musae* | 0.6514 | 58 | *Cronartium ribicola* | 0.4839 | 92 | *Curvularia* | 0.3776 |
| 25 | *Myrothecium roridum* | 0.6509 | 59 | *Lecanicillium lecanii* | 0.4772 | 93 | *Septoria glycines* | 0.3766 |
| 26 | *Magnaporthe salvinii* | 0.6428 | 60 | *Entyloma oryzae* | 0.4692 | 94 | *Mycosphaerella gibsonii* | 0.3621 |
| 27 | *Alternaria longipes* | 0.6353 | 61 | *Physoderma maydis* | 0.4678 | 95 | *Phakopsora euvitis* | 0.3572 |
| 28 | *Peronosclerospora sorghi* | 0.6311 | 62 | *Alternaria padwickii* | 0.4671 | 96 | *Hemileia vastatrix* | 0.3565 |
| 29 | *Fusarium oxysporum f.sp. vasinfectum* | 0.6291 | 63 | *Gibberella acuminata* | 0.4547 | 97 | *Alternaria mali* | 0.3542 |
| 30 | *Aspergillus niger* | 0.6222 | 64 | *Uromyces ciceris-arietini* | 0.4520 | 98 | *Guignardia bidwellii* | 0.3508 |
| 31 | *Cochliobolus miyabeanus* | 0.6209 | 65 | *Septoria cannabis* | 0.4511 | 99 | *Phaeosphaeria avenaria f.sp. avenaria* | 0.3437 |
| 32 | *Gibberella avenacea* | 0.6178 | 66 | *Thanatephorus cucumeris* | 0.4432 | 100 | *Phytophthora vignae* | 0.3434 |
| 33 | *Cercospora kikuchii* | 0.6166 | 67 | *Nattrassia mangiferae* | 0.4419 |  |  |  |
| 34 | *Alternaria radicina* | 0.6128 | 68 | *Cryphonectria parasitica* | 0.4405 |  |  |  |
