# Supplementary material for: Predicting Invasive Fungal Pathogens Using Invasive Pest Assemblages: Testing Model Predictions in a Virtual World
Source: PLoS One. 2011 Oct 10;6(10):e25695. doi: 10.1371/journal.pone.0025695 (PMC3189937; doi:10.1371/journal.pone.0025695)
Supplement: Table S7 — The top 100 list for plant pathogen species absent from the Northern Territory. (DOC) [file pone.0025695.s007.doc]

Table S7. The top 100 list for plant pathogen species absent from the Northern Territory.

| **Rank** | **Species Name** | **Likelihood Index** | **Rank** | **Species Name** | **Likelihood Index** | **Rank** | **Species Name** | **Likelihood Index** |
| --- | --- | --- | --- | --- | --- | --- | --- | --- |
| 1 | *Glomerella tucumanensis* | 0.6302 | 35 | *Didymella bryoniae* | 0.2351 | 69 | *Coleosporium ipomoeae* | 0.1327 |
| 2 | *Ceratocystis paradoxa* | 0.5836 | 36 | *Septoria lycopersici* | 0.2307 | 70 | *Mycosphaerella brassicicola* | 0.1311 |
| 3 | *Corticium salmonicolor* | 0.5162 | 37 | *Marasmius crinis-equi* | 0.2266 | 71 | *Aspergillus niger* | 0.1300 |
| 4 | *Puccinia polysora* | 0.5015 | 38 | *Guignardia musae* | 0.2240 | 72 | *Crinipellis perniciosa* | 0.1280 |
| 5 | *Pseudoperonospora cubensis* | 0.4911 | 39 | *Ustilago zeae* | 0.2220 | 73 | *Corticium koleroga* | 0.1261 |
| 6 | *Hemileia vastatrix* | 0.4580 | 40 | *Colletotrichum gossypii* | 0.2205 | 74 | *Pythium splendens* | 0.1245 |
| 7 | *Uromyces appendiculatus* | 0.4468 | 41 | *Myrothecium roridum* | 0.2084 | 75 | *Nattrassia mangiferae* | 0.1241 |
| 8 | *Mycosphaerella fijiensis* | 0.4236 | 42 | *Puccinia melanocephala* | 0.2073 | 76 | *Pythium vexans* | 0.1227 |
| 9 | *Mycovellosiella fulva* | 0.4117 | 43 | *Thanatephorus cucumeris* | 0.2062 | 77 | *Trachysphaera fructigena* | 0.1213 |
| 10 | *Colletotrichum musae* | 0.4113 | 44 | *Colletotrichum lindemuthianum* | 0.1992 | 78 | *Sarocladium oryzae* | 0.1200 |
| 11 | *Mycosphaerella henningsii* | 0.4079 | 45 | *Gibberella xylarioides* | 0.1926 | 79 | *Colletotrichum acutatum* | 0.1179 |
| 12 | *Phytophthora infestans* | 0.3621 | 46 | *Ceratocystis fimbriata* | 0.1922 | 80 | *Pythium aphanidermatum* | 0.1161 |
| 13 | *Mycosphaerella cruenta* | 0.3406 | 47 | *Puccinia sorghi* | 0.1842 | 81 | *Peronosclerospora sorghi* | 0.1148 |
| 14 | *Alternaria dauci* | 0.3392 | 48 | *Phytophthora citrophthora* | 0.1840 | 82 | *Claviceps fusiformis* | 0.1127 |
| 15 | *Lasiodiplodia theobromae* | 0.3332 | 49 | *Glomerella graminicola* | 0.1777 | 83 | *Taphrina deformans* | 0.1123 |
| 16 | *Nectria rigidiuscula* | 0.3152 | 50 | *Fusarium oxysporum f.sp. vasinfectum* | 0.1681 | 84 | *Didymella lycopersici* | 0.1082 |
| 17 | *Ustilago scitaminea* | 0.3134 | 51 | *Curvularia* | 0.1666 | 85 | *Fusarium oxysporum f.sp. elaeidis* | 0.1074 |
| 18 | *Bipolaris sacchari* | 0.3105 | 52 | *Phytophthora colocasiae* | 0.1665 | 86 | *Sclerospora graminicola* | 0.1065 |
| 19 | *Colletotrichum capsici* | 0.3076 | 53 | *Magnaporthe salvinii* | 0.1664 | 87 | *Bipolaris heveae* | 0.1051 |
| 20 | *Phaeoisariopsis griseola* | 0.3070 | 54 | *Ganoderma philippii* | 0.1651 | 88 | *Botryosphaeria ribis* | 0.1046 |
| 21 | *Phellinus noxius* | 0.2997 | 55 | *Rosellinia pepo* | 0.1642 | 89 | *Cryphonectria cubensis* | 0.1037 |
| 22 | *Mycosphaerella arachidis* | 0.2975 | 56 | *Sphacelotheca reiliana* | 0.1584 | 90 | *Stenocarpella macrospora* | 0.1028 |
| 23 | *Gloeocercospora sorghi* | 0.2961 | 57 | *Sporisorium cruentum* | 0.1569 | 91 | *Tilletia barclayana* | 0.1023 |
| 24 | *Macrophomina phaseolina* | 0.2875 | 58 | *Mycovellosiella koepkei* | 0.1565 | 92 | *Fusarium oxysporum* | 0.1021 |
| 25 | *Rigidoporus microporus* | 0.2867 | 59 | *Diplocarpon rosae* | 0.1538 | 93 | *Marasmiellus scandens* | 0.1014 |
| 26 | *Cercospora beticola* | 0.2866 | 60 | *Cercospora elaeidis* | 0.1537 | 94 | *Ascochyta sorghi* | 0.1000 |
| 27 | *Rosellinia bunodes* | 0.2645 | 61 | *Puccinia kuehnii* | 0.1457 | 95 | *Puccinia triticina* | 0.0984 |
| 28 | *Alternaria porri* | 0.2644 | 62 | *Plasmopara viticola* | 0.1452 | 96 | *Puccinia graminis* | 0.0978 |
| 29 | *Sporisorium sorghi* | 0.2527 | 63 | *Mycosphaerella citri* | 0.1423 | 97 | *Sclerotinia sclerotiorum* | 0.0968 |
| 30 | *Cladosporium musae* | 0.2503 | 64 | *Cercospora nicotianae* | 0.1414 | 98 | *Bipolaris incurvata* | 0.0957 |
| 31 | *Elsino batatas* | 0.2477 | 65 | *Mycosphaerella coffeicola* | 0.1381 | 99 | *Cercospora caribaea* | 0.0939 |
| 32 | *Alternaria brassicicola* | 0.2464 | 66 | *Mycena citricolor* | 0.1379 | 100 | *Cochliobolus sativus* | 0.0939 |
| 33 | *Colletotrichum truncatum* | 0.2450 | 67 | *Phaeoramularia angolensis* | 0.1335 |  |  |  |
| 34 | *Leveillula taurica* | 0.2374 | 68 | *Albugo candida* | 0.1329 |  |  |  |
