# Supplementary material for: Predicting Invasive Fungal Pathogens Using Invasive Pest Assemblages: Testing Model Predictions in a Virtual World
Source: PLoS One. 2011 Oct 10;6(10):e25695. doi: 10.1371/journal.pone.0025695 (PMC3189937; doi:10.1371/journal.pone.0025695)
Supplement: Table S8 — The top 100 list for plant pathogen species absent from Tasmania. (DOC) [file pone.0025695.s008.doc]

Table S8. The top 100 list for plant pathogen species absent from Tasmania.

| **Rank** | **Species Name** | **Likelihood Index** | **Rank** | **Species Name** | **Likelihood Index** | **Rank** | **Species Name** | **Likelihood Index** |
| --- | --- | --- | --- | --- | --- | --- | --- | --- |
| 1 | *Phytophthora infestans* | 0.7975 | 35 | *Kabatiella caulivora* | 0.4111 | 69 | *Glomerella cingulata* | 0.3172 |
| 2 | *Gibberella avenacea* | 0.6834 | 36 | *Puccinia helianthi* | 0.4078 | 70 | *Rhizina undulata* | 0.3158 |
| 3 | *Cercospora beticola* | 0.6715 | 37 | *Leveillula taurica* | 0.4050 | 71 | *Rhabdocline pseudotsugae* | 0.3097 |
| 4 | *Pseudoperonospora humuli* | 0.6608 | 38 | *Stemphylium sarciniforme* | 0.3993 | 72 | *Lachnella willkommii* | 0.3093 |
| 5 | *Alternaria brassicicola* | 0.6592 | 39 | *Peronospora destructor* | 0.3987 | 73 | *Ceratobasidium cereale* | 0.3085 |
| 6 | *Cronartium ribicola* | 0.6548 | 40 | *Colletotrichum acutatum* | 0.3901 | 74 | *Plasmopara halstedii* | 0.3041 |
| 7 | *Gibberella zeae* | 0.6341 | 41 | *Cronartium flaccidum* | 0.3862 | 75 | *Polyscytalum pustulans* | 0.3000 |
| 8 | *Venturia pirina* | 0.6242 | 42 | *Didymella lycopersici* | 0.3831 | 76 | *Hypoxylon mammatum* | 0.2999 |
| 9 | *Albugo candida* | 0.6030 | 43 | *Didymella bryoniae* | 0.3828 | 77 | *Didymella lentis* | 0.2976 |
| 10 | *Ustilago zeae* | 0.5865 | 44 | *Pezicula malicorticis* | 0.3782 | 78 | *Colletotrichum linicola* | 0.2948 |
| 11 | *Puccinia asparagi* | 0.5784 | 45 | *Nectria cinnabarina* | 0.3679 | 79 | *Ramularia onobrychidis* | 0.2924 |
| 12 | *Peronospora hyoscyami f.sp. tabacina* | 0.5727 | 46 | *Peronospora manshurica* | 0.3673 | 80 | *Venturia cerasi* | 0.2900 |
| 13 | *Heterobasidion annosum* | 0.5636 | 47 | *Septoria cannabis* | 0.3613 | 81 | *Cryphonectria parasitica* | 0.2896 |
| 14 | *Puccinia allii* | 0.5471 | 48 | *Blumeria graminis* | 0.3533 | 82 | *Fomes fomentarius* | 0.2874 |
| 15 | *Podosphaera aphanis* | 0.5461 | 49 | *Melampsora medusae* | 0.3512 | 83 | *Blumeriella jaapii* | 0.2844 |
| 16 | *Puccinia triticina* | 0.5329 | 50 | *Alternaria radicina* | 0.3504 | 84 | *Pleiochaeta setosa* | 0.2795 |
| 17 | *Pyrenophora graminea* | 0.5326 | 51 | *Lophodermium pinastri* | 0.3462 | 85 | *Ophiostoma piceae* | 0.2766 |
| 18 | *Pyrenophora tritici-repentis* | 0.5315 | 52 | *Chrysomyxa abietis* | 0.3458 | 86 | *Armillaria ostoyae* | 0.2766 |
| 19 | *Ceratocystis ulmi* | 0.5270 | 53 | *Gymnosporangium fuscum* | 0.3438 | 87 | *Guignardia bidwellii* | 0.2761 |
| 20 | *Monilinia fructigena* | 0.5240 | 54 | *Meria laricis* | 0.3428 | 88 | *Didymascella thujina* | 0.2724 |
| 21 | *Podosphaera pannosa* | 0.5184 | 55 | *Fusarium oxysporum f.sp. lini* | 0.3398 | 89 | *Gnomonia comari* | 0.2660 |
| 22 | *Gibberella fujikuroi* | 0.5073 | 56 | *Peronospora farinosa* | 0.3365 | 90 | *Colletotrichum orbiculare* | 0.2565 |
| 23 | *Sporisorium sorghi* | 0.5023 | 57 | *Tilletia controversa* | 0.3363 | 91 | *Fomitopsis pinicola* | 0.2540 |
| 24 | *Helicobasidium brebissonii* | 0.4948 | 58 | *Alternaria porri* | 0.3332 | 92 | *Puccinia carthami* | 0.2539 |
| 25 | *Podosphaera macularis* | 0.4903 | 59 | *Venturia inaequalis* | 0.3321 | 93 | *Magnaporthe salvinii* | 0.2482 |
| 26 | *Urocystis cepulae* | 0.4896 | 60 | *Cladosporium cucumerinum* | 0.3318 | 94 | *Physoderma alfalfae* | 0.2459 |
| 27 | *Phytophthora fragariae* | 0.4877 | 61 | *Synchytrium endobioticum* | 0.3288 | 95 | *Mycosphaerella pyri* | 0.2448 |
| 28 | *Pseudoperonospora cubensis* | 0.4645 | 62 | *Pyrenophora teres* | 0.3268 | 96 | *Magnaporthe grisea* | 0.2438 |
| 29 | *Mycosphaerella linicola* | 0.4485 | 63 | *Pyrenophora chaetomioides* | 0.3229 | 97 | *Sclerophthora macrospora* | 0.2437 |
| 30 | *Drepanopeziza ribis* | 0.4432 | 64 | *Puccinia sorghi* | 0.3225 | 98 | *Pythium graminicola* | 0.2410 |
| 31 | *Fusarium oxysporum* | 0.4366 | 65 | *Aphanomyces euteiches* | 0.3224 | 99 | *Fusarium oxysporum f.sp. melonis* | 0.2335 |
| 32 | *Erysiphe necator* | 0.4269 | 66 | *Phialophora cinerescens* | 0.3198 | 100 | *Cryptodiaporthe populea* | 0.2319 |
| 33 | *Gremmeniella abietina* | 0.4168 | 67 | *Phaeolus schweinitzii* | 0.3198 |  |  |  |
| 34 | *Sphacelotheca reiliana* | 0.4130 | 68 | *Entyloma dahliae* | 0.3186 |  |  |  |
