# Supplementary material for: Predicting Invasive Fungal Pathogens Using Invasive Pest Assemblages: Testing Model Predictions in a Virtual World
Source: PLoS One. 2011 Oct 10;6(10):e25695. doi: 10.1371/journal.pone.0025695 (PMC3189937; doi:10.1371/journal.pone.0025695)
Supplement: Table S9 — Full list of neurons and associated regions from the SOM analysis of fungal pathogen assemblages. (DOC) [file pone.0025695.s009.doc]

Table S9. Full list of neurons and associated regions from the SOM analysis of fungal pathogen assemblages.

| **Neuron** | **Region** |
| --- | --- |
| 1 | Canada, Germany, France, United Kingdom, Italy, New Zealand, USA |
| 2 |  |
| 3 | Argentina, Australia, New South Wales (Australia), China, India, Japan, Korea- Republic of, South Africa |
| 4 | Pakistan |
| 5 | Queensland (Australia), Brazil, Taiwan, Colombia, Kenya, Mexico, Tanzania, Zimbabwe |
| 6 | Malawi, Uganda, Florida (USA), Venezuela, Zambia |
| 7 | Costa Rica, Cuba, Guatemala, Jamaica, Malaysia, Nigeria, Panama, Papua New Guinea, Philippines, Puerto Rico, Thailand, Hawaii (USA) |
| 8 | Mauritius, El Salvador |
| 9 | Dominican Republic, Fiji, Ghana, Guyana, Honduras, Haiti, Indonesia, Sri Lanka, Madagascar, Peninsular Malaysia, Sabah (Malaysia), Nicaragua, Trinidad and Tobago, Congo Democratic Republic |
| 10 | New Caledonia, Vietnam |
| 11 | Barbados, Brunei Darussalam, Côte d'Ivoire, Cambodia, Sarawak (Malaysia), Sierra Leone, Senegal, Suriname |
| 12 |  |
| 13 | American Samoa, Cameroon, Gabon Guinea, Java (Indonesia), Solomon Islands, Togo, Samoa |
| 14 | Austria, Bulgaria, Hungary, Netherlands, Poland, Romania, Russian Federation |
| 15 | Spain, Greece |
| 16 | Israel, Iran, Turkey |
| 17 | Egypt |
| 18 | Ethiopia |
| 19 | Bolivia, Nepal |
| 20 | Mozambique |
| 21 | Bangladesh, Myanmar, Sudan |
| 22 |  |
| 23 | Angola |
| 24 |  |
| 25 | Australian Northern Territory (Australia) |
| 26 | Belize, Congo, Dominica, French Polynesia, Tonga, Saint Vincent and the Grenadines, Vanuatu |
| 27 | Switzerland, Czechoslovakia (former -), Denmark, Norway, Sweden |
| 28 |  |
| 29 | Chile, Morocco, Portugal |
| 30 | Cyprus, Iraq, Lebanon, Uruguay |
| 31 | Peru |
| 32 |  |
| 33 | Sao Paulo (Brazil) |
| 34 | Karnataka (India), Maharashtra (India), Tamil Nadu (India) |
| 35 | Hong Kong (China) |
| 36 | Bermuda |
| 37 | Ecuador, Laos, Niger, Somalia |
| 38 | Benin, Central African Republic, Irian Jaya (Indonesia) |
| 39 | Grenada, Guadeloupe, Guam, Sumatra (Indonesia), Kerala (India), Saint Lucia, Martinique |
| 40 | Belgium, Finland, Ireland, Ukraine, Yugoslavia |
| 41 | Tasmania (Australia), Lithuania, Latvia |
| 42 | South Australia (Australia), Victoria (Australia), Western Australia (Australia), Former Yugoslavia |
| 43 | Korea- DPR, Libya |
| 44 | Afghanistan, Rio Grande do Sul (Brazil), Saudi Arabia |
| 45 |  |
| 46 | Indian Punjab (India), Uttar Pradesh (India) |
| 47 | Bihar (India), Haryana (India), Jammu and Kashmir (India), Madhya Pradesh (India), Rajasthan (India), West Bengal (India) |
| 48 | Minas Gerais (Brazil), Andhra Pradesh (India), Assam (India) |
| 49 | Bhutan, Yemen |
| 50 | Burkina Faso, Gambia, Mali, Paraguay, Rwanda, Singapore, Chad |
| 51 |  |
| 52 | Antigua and Barbuda, Federated states of Micronesia, French Guiana, Saint Kitts and Nevis, Réunion |
| 53 | British Columbia (Canada), Ontario (Canada), England and Wales |
| 54 |  |
| 55 | Russia (Europe) (Russian Federation) |
| 56 | Kazakhstan, Russian Far East (Russian Federation) |
| 57 |  |
| 58 | Guangxi (China), Henan (China), Jiangsu (China), Jiangxi (China), Liaoning (China), Sichuan (China), Yunnan (China), Zhejiang (China) |
| 59 | Guangdong (China) |
| 60 | Himachal Pradesh (India) |
| 61 | Delhi (India), Gujarat (India), Orissa (India) |
| 62 | Meghalaya (India), Sikkim (India), Ryukyu Archipelago (Japan) |
| 63 | Burundi, Botswana, Canary Islands (Spain), Swaziland |
| 64 | Mauritania |
| 65 | Bahia (Brazil), Espirito Santo (Brazil), Pernambuco (Brazil), Cook Islands, Kalimantan (Indonesia), Nusa Tenggara (Indonesia), Andaman and Nicobar Islands (India), Liberia |
| 66 | Alberta (Canada), Manitoba (Canada), New Brunswick (Canada), Nova Scotia (Canada), Quebec (Canada), Saskatchewan (Canada), California (USA), Idaho (USA), Oregon (USA), Washington (USA) |
| 67 |  |
| 68 | Prince Edward Island (Canada) |
| 69 |  |
| 70 |  |
| 71 | Anhui (China), Gansu (China), Hebei (China), Heilongjiang (China), Jilin (China), Shandong (China), Shanxi (China), Shaanxi (China) , Honshu (Japan) |
| 72 | Fujian (China), Guizhou (China), Hubei (China), Hunan (China) |
| 73 |  |
| 74 | Manipur (India) |
| 75 | Parana (Brazil), Nagaland (India), Kyushu (Japan), Shikoku (Japan) |
| 76 | Goias (Brazil), Matto Grosso (Brazil), Santa Catarina (Brazil), Hainan (China), Oman |
| 77 | Acre (Brazil), Rondonia (Brazil), United States Virgin Islands |
| 78 | Amazonas (Brazil), Ceara (Brazil), Pará (Brazil), Rio de Janeiro (Brazil), Equatorial Guinea, Moluccas (Indonesia), Sulawesi (Indonesia), Cayman Islands, Northern Mariana Islands, Montserrat, Norfolk Island, Niue, Seychelles, Sao Tome and Principe |
| 79 | Minnesota (USA), New York (USA), Wisconsin (USA) |
| 80 | Pennsylvania (USA) |
| 81 | Connecticut (USA), Massachusetts (USA), Michigan (USA) |
| 82 | Maine (USA) |
| 83 |  |
| 84 |  |
| 85 | Nei Menggu (China), Xinjiang (China) |
| 86 | Hokkaido (Japan) |
| 87 | Ningxia (China), Qinghai (China), Xizhang (China) |
| 88 |  |
| 89 | United Arab Emirates, Matto Grosso do Sul (Brazil), Chandigarh (India), Mizoram (India), Tripura (India) |
| 90 | Lesotho |
| 91 | Netherlands Antilles, Piauí (Brazil), Bahamas, Cape Verde, Guinea-Bissau, Arunachal Pradesh (India), Lakshadweep (India), Kiribati, Comoros, Rodriguez Island (Muritius), Belau, Tuvalu, Wallis and Futuna |
| 92 | Georgia (USA), Iowa (USA), Illinois (USA), Indiana (USA), Maryland (USA), Mississippi (USA), North Carolina (USA), Ohio (USA), Oklahoma (USA), Texas (USA), Virginia (USA) |
| 93 | Alabama (USA), Arkansas (USA), Kansas (USA), Kentucky (USA), Louisiana (USA), Missouri (USA), South Carolina (USA), Tennessee (USA) |
| 94 | Delaware (USA), Nebraska (USA), New Jersey (USA) , South Dakota (USA), West Virginia (USA) |
| 95 | Arizona (USA), Colorado (USA), Montana (USA), North Dakota (USA), New Mexico (USA), Utah (USA), Wyoming (USA) |
| 96 | Alaska (USA) |
| 97 | Armenia, Azerbaijan, Czech Republic, Algeria, Estonia, Georgia (Republic), Moldova, Central Russia, Slovakia, Syria, Tunisia, Uzbekistan |
| 98 | Siberia (Russian Federation) |
| 99 | Belarus, Northern Ireland, Scotland, Croatia, Jordan, Kyrgyzstan, Malta, Southern Russia (Russian Federation), Slovenia, Turkmenistan |
| 100 | Channel Islands (UK), Crete, Mongolia |
| 101 | Albania, Bosnia and Herzegovina, Newfoundland (Canada), Corsica (France), Iceland, Sardinia (Italy), Luxembourg, Macedonia, Azores (Portugal), Western Siberia, Former USSR, Tajikistan, New Hampshire (USA), Nevada (USA), Rhode Island (USA), Vermont (USA) |
| 102 | Sicily (Italy), Madeira (Portugal) |
| 103 | Andorra, Northwest Territories (Canada), Yukon Territory (Canada), Balearic Islands (Spain), Falkland Islands, Faroe Islands, Greenland, Gaza, Liechtenstein, Monaco, Namibia, Russia (Asia) (Russian Federation), Eastern Siberia (Russian Federation), Northern Russia (Russian Federation), San Marino, Krymskaya Oblast (Ukraine), District of Columbia (USA), Serbia |
| 104 | Bahrain, Alagoas (Brazil), Amapa (Brazil), Maranhao (Brazil), Paraiba (Brazil), Rio Grande do Norte (Brazil), Roraima (Brazil), Cocos Islands, Beijing (China), Shanghai (China), Curaçao, Christmas Island (Indian Ocean), Eritrea, Caroline Islands (Micronesia), Guernsey, Gibraltar, Goa (India), British Indian Ocean Territory, Kanton and Enderbury (Kiribati), Kuwait, Marshall Islands, Maldives, Saint Pierre and Miquelon, Pitcairn Islands, Saint Helena, East Timor, Zanzibar (Tanzania), British Virgin Islands |
