# Supplementary material for: Predicting Invasive Fungal Pathogens Using Invasive Pest Assemblages: Testing Model Predictions in a Virtual World
Source: PLoS One. 2011 Oct 10;6(10):e25695. doi: 10.1371/journal.pone.0025695 (PMC3189937; doi:10.1371/journal.pone.0025695)
Supplement: Table S10 — Acceptability of lists generated for regions with less than 8 species present. (DOC) [file pone.0025695.s010.doc]

Table S10. Acceptability of lists generated for regions with less than 8 species present.

| **Region** | **# species** | **# regions in neuron^1^** | **accept risk list** |  | **Region** | **# species** | **# regions in neuron^1^** | **accept risk list** |
| --- | --- | --- | --- | --- | --- | --- | --- | --- |
| Amapa | 1 | n/a | No |  | Mizoram | 3 | 5 | No |
| Roraima | 1 | n/a | No |  | Liechtenstein | 3 | 18 | Yes |
| Curaçao | 1 | n/a | No |  | Tuvalu | 3 | 13 | Yes |
| Gibraltar | 1 | n/a | No |  | Andorra | 4 | 18 | Yes |
| British Indian Ocean Territory | 1 | n/a | No |  | Guernsey | 4 | 28 | Yes |
| Kiribati | 1 | n/a | No |  | Guinea-Bissau | 4 | 13 | Yes |
| Kanton and Enderbury | 1 | n/a | No |  | Zanzibar | 4 | 28 | Yes |
| Monaco | 1 | n/a | No |  | Acre | 5 | 3 | No |
| Marshall Islands | 1 | n/a | No |  | Maranhao | 5 | 28 | Yes |
| Maldives | 1 | n/a | No |  | Paraiba | 5 | 28 | Yes |
| Saint Pierre and Miquelon | 1 | n/a | No |  | Shanghai | 5 | 28 | Yes |
| Pitcairn Islands | 1 | n/a | No |  | Falkland Islands | 5 | 18 | Yes |
| San Marino | 1 | n/a | No |  | Arunachal Pradesh | 5 | 13 | Yes |
| East Timor | 1 | n/a | No |  | Lakshadweep | 5 | 13 | Yes |
| Bahrain | 2 | n/a | No |  | Tripura | 5 | 5 | No |
| Alagoas | 2 | n/a | No |  | District of Columbia | 5 | 18 | Yes |
| Rio Grande do Norte | 2 | n/a | No |  | Rondonia | 6 | 3 | No |
| Cocos Islands | 2 | n/a | No |  | Moluccas | 6 | 14 | Yes |
| Beijing | 2 | n/a | No |  | Nagaland | 6 | 4 | Yes |
| Cape Verde | 2 | n/a | No |  | Lesotho | 6 | 1 | No |
| Greenland | 2 | n/a | No |  | Montserrat | 6 | 14 | Yes |
| Gaza | 2 | n/a | No |  | Russia (Asia) | 6 | 18 | Yes |
| Chandigarh | 2 | n/a | No |  | Krymskaya Oblast | 6 | 18 | Yes |
| Goa | 2 | n/a | No |  | Serbia | 6 | 18 | Yes |
| Rodriguez Island | 2 | n/a | No |  | Piauí | 7 | 13 | Yes |
| Namibia | 2 | n/a | No |  | Bahamas | 7 | 13 | Yes |
| Saint Helena | 2 | n/a | No |  | Equatorial Guinea | 7 | 14 | Yes |
| British Virgin Islands | 2 | n/a | No |  | Comoros | 7 | 13 | Yes |
| Christmas Island | 3 | 28 | Yes |  | Kuwait | 7 | 28 | Yes |
| Eritrea | 3 | 28 | Yes |  | Northern Mariana Islands | 7 | 14 | Yes |
| Balearic Islands | 3 | 18 | Yes |  | Wallis and Futuna | 7 | 13 | Yes |
| Caroline Islands | 3 | 28 | Yes |  |  |  |  |  |
